# Climate change did not alter the effects of Bt maize on soil Collembola in northeast China

**DOI:** 10.1038/s41598-022-16783-2

**Published:** 2022-08-04

**Authors:** Baifeng Wang, Junqi Yin, Fengci Wu, Daming Wang, Zhilei Jiang, Xinyuan Song

**Affiliations:** Jilin Provincial Key Laboratory of Agricultural Biotechnology, Agro-Biotechnology Research Institute, Jilin Academy of Agriculture Sciences, Changchun, 130033 China

**Keywords:** Climate sciences, Ecology, Environmental sciences

## Abstract

Bt maize is being increasingly cultivated worldwide as the effects of climate change are increasing globally. Bt maize IE09S034 and its near-isogenic non-Bt maize Zong 31 were used to investigate whether climate change alters the effects of Bt maize on soil Collembola. Warming and drought conditions were simulated using open-top chambers (OTC), and their effects on soil Collembola were evaluated. We found that the maize type had no significant effect on Collembola; however, the abundance and diversity of Collembola were significantly higher in the OTC than outside at the seedling stage; they were significantly lower in the OTC at the heading and mature stages. The interactions of the maize type with the OTC had no effect on these parameters. Therefore, Bt maize had no significant effect on soil Collembola, and the effects of climate warming and drought on soil Collembola depended on the ambient climatic conditions. When the temperature was low, collembolan abundance and diversity were promoted by warming; however, when the temperature was high and the humidity was low, collembolan abundance and diversity were inhibited by warming and drought. The climate changes simulated by the OTC did not alter the effects of Bt maize on soil Collembola.

## Introduction

Bt maize varieties are being increasingly grown worldwide because they can reduce the use of pesticides, have enormous environmental and human health benefits, and help improve the economic conditions of farmers^1^. Bt protein from these maize varieties can form micro aggregates in soil and remain active for several months^2–6^, which may affect the soil fauna^7–9^.

Temperature and humidity are the two most important climate factors, and changes in these could significantly influence the function and services provided by an ecosystem^10–12^. Warming, combined with drought, has occurred frequently during the past several decades worldwide^13–15^, and has affected ecosystems in many ways^16–19^. One of the most notable impacts of warming and droughts is their impact on the soil ecosystem^20–22^. Warming and drought can indirectly affect the soil ecosystem by affecting the aboveground plants; for example, they can alter the biosynthesis of lignin and increase its quantity^23^, reduce photosynthesis^24^, increase the N/P ratio, and decrease the total aboveground plant biomass growth^25^. These effects may, in turn, affect the underground ecosystem by changing the organic matter input. Warming combined with drought can also directly affect the underground soil ecosystem by affecting the underground microorganisms^26^, the functional enzyme activities^27^, and the soil fauna^28,29^. Warming and drought have occurred simultaneously in the past^15,30^ and if global temperatures continue to rise at the current rate, global warming is projected to reach 1.5 °C between the years 2030 and 2052^31^. Consequently, the future agro-ecosystems may be seriously impacted by climate change. Will warming and drought alter the growth of Bt maize crops root and the soil ecosystems (including the soil fauna) surrounding them? To the best of our knowledge, no existing literature has addressed this question.

Collembola are some of the most ubiquitous and abundant members of the soil mesofauna^32,33^. They play an important role in material circulation and energy transformation in the soil ecosystem^34–36^. Additionally, Collembola are sensitive to environmental changes; thus, they have been widely used as indicators for evaluating environmental pollution in soil^37–39^. With increasing cultivation of Bt maize, Collembola have been used for evaluating the potential impacts of Bt maize cultivation on the soil environment^40–42^. Furthermore, Collembola live in different soil layers, usually with different tolerance to warming and desiccation. For example, Symphypleona (species living in upper soil layers) have developed a tracheal system and continuous lipid/wax layer of cuticle (high impermeability of cuticle), and some other possible adaptations, so they have a better ability to survive of drought; while the soil-dwelling Arthropleona do not have such specialized adaptations, consequently, they are highly sensitive to desiccation in general^43,44^. Therefore, studying the response of Collembola at species level to Bt maize under the predicted climate future conditions of warming and drought will be helpful for scientifically evaluating the potential ecological risk of Bt crops to soil ecosystems under future climate change conditions.

In the present study, Bt maize IE09S034 and its near-isogenic non-Bt maize Zong 31 were used as the experimental crops, and open-top chambers (OTCs) were used to simulate warming and drought, which are expected with climate change. We analyzed the effects of the maize type, OTC treatment, and sampling stage on the abundance and diversity of Collembola. Our study aimed to clarify whether warming combined with drought will alter the effects of Bt maize cultivation on soil Collembola. We hypothesized that (1) Bt maize would have no significant effect on the soil collembolan community; (2) climate change simulated by OTC would have a significant effect on the soil collembolan community; and (3) climate change simulated by OTC would alter the effects of Bt maize on soil Collembola.

## Results

### Soil parameters

**Figure 1:**
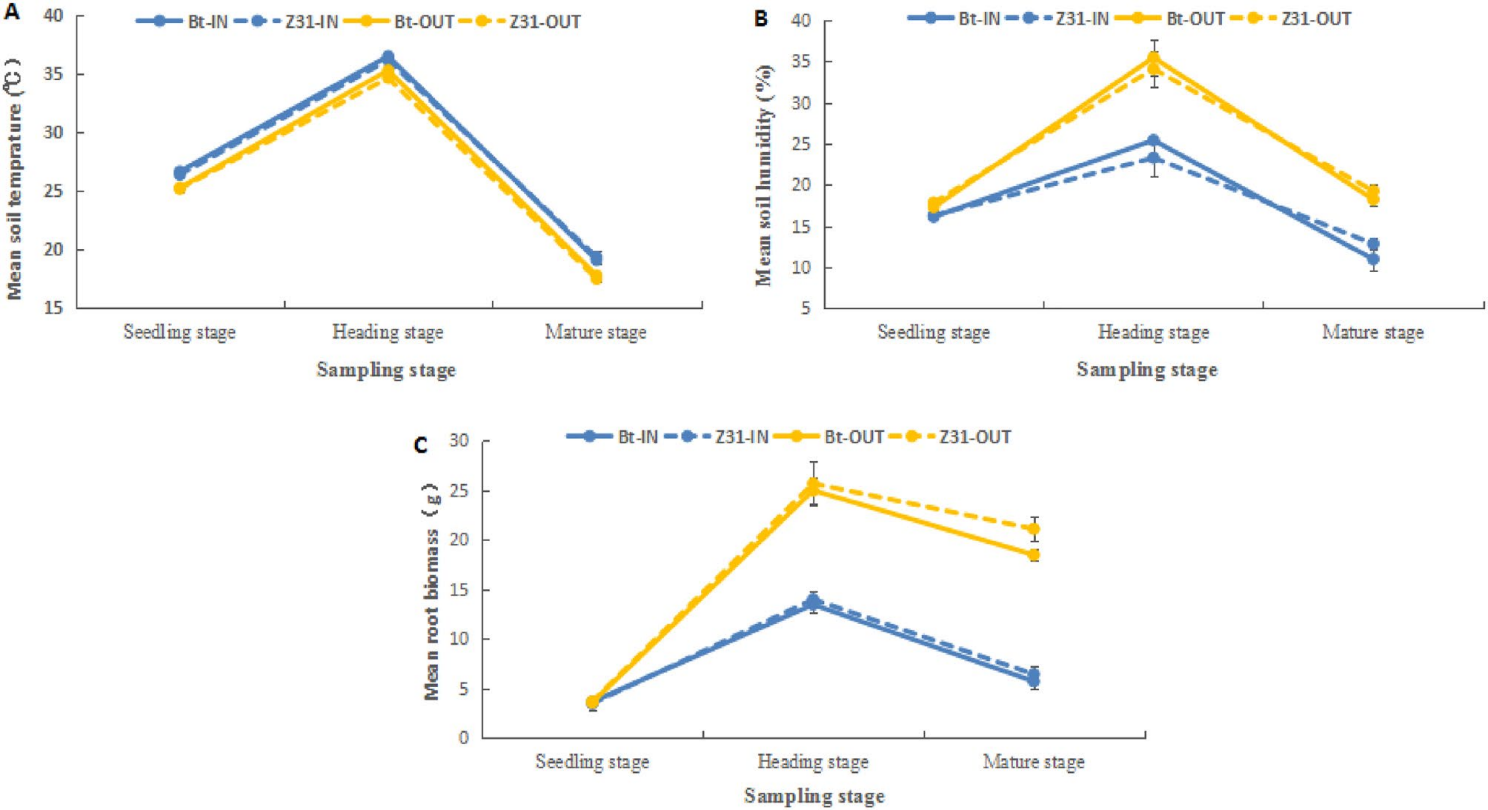
The mean soil temperature (**A**), soil humidity (**B**), and maize root biomass (**C**) of the different experimental treatment plots at different sampling stages.

The mean soil temperature was approximately 25 °C at the seedling stage, increased to approximately 35 °C at the heading stage, and then decreased to approximately 18 °C at the mature stage. One-way analysis of variance (ANOVA) showed that for the same maize type, the mean soil temperature was ~ 1 °C higher in OTC than outside for all the sampling stages (df = 1, 7; *P* < 0.05). The soil humidity and root biomass were low at the seedling stage and were not significantly different between the OTC and outside (df = 1, 7; *P* > 0.05). However, with time, the soil humidity and root biomass in OTC were both significantly lower than those observed outside at the heading and mature stages (df = 1, 7; *P* < 0.05) (Fig. 1).

### Collembolan community composition

A total of 19,581 individuals belonging to 7 families, 13 genera, and 17 species of Collembola were sampled. In total, 4561 individuals were in Bt maize in OTC, 4660 were in non-Bt maize Zong 31 in OTC, 5141 were in Bt maize outside OTC, and 5219 were in non-Bt maize Zong 31 outside OTC. The dominant species in all treatments was *Hypogastrura yosii* (52.84% in the Bt maize in OTC, 49.27% in the non-Bt maize in OTC, 43.18% in the Bt maize outside OTC, and 44.93% in the non-Bt maize outside OTC) and *Thalassaphorura macrospinata* (14.84% in the Bt maize in OTC, 18.37% in the non-Bt maize in OTC, 25.77% in the Bt maize outside OTC, and 26.00% in the non-Bt maize outside OTC). Other relatively abundant species were *Folsomides parvulus, Isotomodes* sp., and *Entomobrya* sp.4, with dominance more than 3% and less than 10% in each treatment. The remaining species were relatively rare (Table 1).

**Table 1:** Number of specimens (N) and dominance (D) of soil Collembola captured in the maize fields that received different experimental treatments.

Species	Bt-IN		Z31-IN		Bt-OUT		Z31-OUT	
	N	D (%)	N	D (%)	N	D (%)	N	D (%)
*Thalassaphorura macrospinata* Sun &Wu, 2012	677	14.84	856	18.37	1325	25.77	1357	26.00
*Folsomides parvulus* Stach, 1922	373	8.18	450	9.66	341	6.63	187	3.58
*Isotomodes* sp.	154	3.38	307	6.59	211	4.10	262	5.02
*Isotomiella minor* (Schäffer, 1896)	8	0.18	2	0.04	3	0.06	12	0.23
*Folsomia bisetosa* Gisin, 1953	98	2.15	81	1.74	182	3.54	222	4.25
*Proisotoma minuta* (Tullberg, 1871)	46	1.01	28	0.6	343	6.67	290	5.56
*Desoria* sp.	100	2.19	59	1.27	34	0.66	24	0.46
*Hypogastrura yosii* Stach, 1964	2410	52.84	2296	49.27	2220	43.18	2345	44.93
*Entomobrya* sp.1	19	0.42	16	0.34	36	0.70	28	0.54
*Entomobrya* sp.2	29	0.64	38	0.82	60	1.17	52	1.00
*Entomobrya* sp.3	46	1.01	41	0.88	17	0.33	49	0.94
*Entomobrya* sp.4	395	8.66	298	6.39	163	3.17	177	3.39
*Orchesellides* sp.1	60	1.32	64	1.37	43	0.84	55	1.05
*Orchesellides* sp.2	0	0.00	5	0.11	5	0.10	5	0.10
*Mesaphorura yosii* (Rusek, 1967)	36	0.79	50	1.07	33	0.64	53	1.02
*Sminthurinus* sp.	55	1.21	36	0.77	40	0.78	31	0.59
*Sminthurides* sp.	55	1.21	33	0.71	85	1.65	72	1.38
Total	4561	100.00	4660	100.00	5141	100.00	5219	100.00

For treatments see the “Methods” section.

### Impacts of maize type, OTC, and sampling stage on Collembola

**Figure 2:**
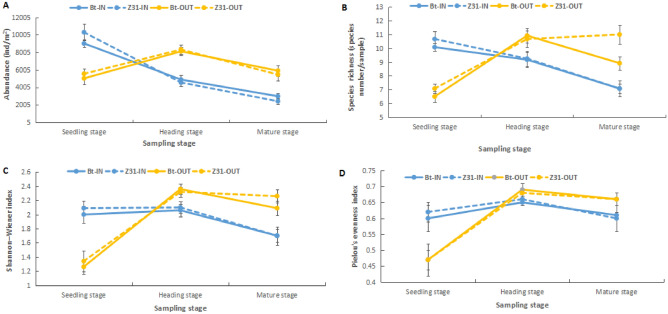
Soil collembolan abundance (**A**), species richness (**B**), Shannon–Wiener index (**C**), and Pielou’s evenness index (**D**) of the different experimental treatment plots at different sampling stages, analyzed using one-way analysis of variance (ANOVA).

The three-way ANOVA results showed that neither the maize type nor the maize type with other factors (OTC and sampling stage) had a significant effect on the collembolan abundance and diversity, indicating that transgenic *cry1Ie* maize did not influence the soil collembolan community. The sampling stage had a significant effect on the collembolan abundance and diversity. The OTC had no significant effect on the collembolan diversity, but had a significant effect on the collembolan abundance, and the interactions of the OTC with the sampling stage had a significant effect on the collembolan abundance and diversity (Table 2). Further ANOVA results showed that for the same maize type, the collembolan abundance, species richness, Shannon–Wiener index, and Pielou’s evenness index were all significantly higher in OTC than outside at the seedling stage; however, all these parameters were significantly lower in OTC than outside at the heading and mature stages (Fig. 2), indicating that the effect of OTC on Collembola was related to the sampling stage, when the temperature is low (seedling stage), Collembola are promoted by OTC, and when the temperature is high and the humidity is low (heading and mature stages), Collembola are inhibited by OTC.

**Table 2:** Effects of the maize type, open-top chambers (OTC), and sampling stage on the soil collembolan abundance, and diversity indices at different sampling stages, as analyzed using three-way analysis of variance (ANOVA).

Variable	Abundance		Species richness		Shannon–Wiener index		Pielou’s evenness index	
	*F*	*P*	*F*	*P*	*F*	*P*	*F*	*P*
Maize type	0.113	0.737	3.124	0.079	1.077	0.301	0.023	0.881
OTC	4.589	0.034*	1.006	0.318	0.013	0.908	1.428	0.234
Sampling stage	34.830	< 0.001***	11.037	< 0.001***	28.273	< 0.001***	25.626	< 0.001***
Maize type × OTC	0.001	0.972	1.006	0.318	0.055	0.814	0.062	0.803
Maize type × sampling stage	1.593	0.207	1.262	0.287	0.281	0.756	0.080	0.923
OTC × sampling stage	59.675	< 0.001***	46.047	< 0.001***	41.707	< 0.001***	15.986	< 0.001***
Maize type × OTC × sampling stage	0.330	0.719	1.691	0.188	0.409	0.665	0.026	0.974

The values highlighted in bold are statistically significant (*P < 0.05; ***P < 0.001).

### Relationship between collembolan communities and environmental variables

**Figure 3:**
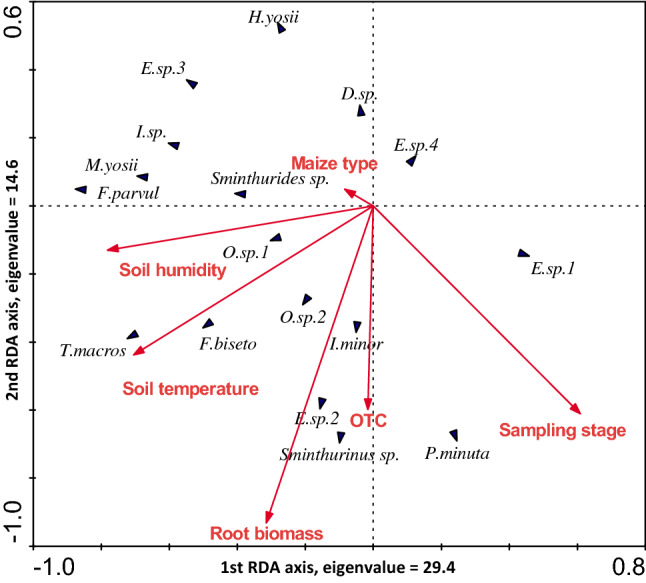
Redundancy analysis (RDA) for indicating associations of collembolan communities with environmental variables.

Redundancy analysis showed that the variables that we selected (including the soil humidity, root biomass, sampling stage, OTC, soil temperature, and maize type) explained 59% of the variation in the collembolan community composition. Among these variables, the soil humidity (21%) explained most of the variation, followed by the root biomass (14%), sampling stage (12%) and OTC (10%). These variables were significantly correlated with the collembolan community composition (Monte Carlo test, *F* = 12.37, 9.52, 9.50, and 10.17, respectively; *P* = 0.002, 0.002, 0.002, and 0.002, respectively), while the soil temperature (1%) and maize variety (1%) were not significantly correlated with the collembolan community composition (*F* = 1.77 and 1.00, respectively; *P* = 0.052 and 0.416, respectively) (Fig. 3, Table 3).

**Table 3:** Effects of soil humidity, root biomass, sampling stage, soil temperature and maize type on the soil Collembola in a Monte Carlo redundancy analysis (RDA) test.

Environmental factor	Variance explained (%)	*F*	*P*
Soil humidity	21	12.37	0.002**
Root biomass	14	9.52	0.002**
Sampling stage	12	9.50	0.002**
OTC	10	10.17	0.002**
Soil temperature	1	1.77	0.052
Maize type	1	1.00	0.416

The values highlighted in bold are statistically significant (**P < 0.01).

## Discussion

We investigated the impact of the maize type, climate change, and sampling stage on the collembolan abundance and diversity. Our results suggest that Bt maize had no significant effect on the soil collembolan community. This is in line with our hypothesis (1) that Bt maize would have no significant effect on the soil collembolan community. Similarly, this is consistent with the findings of our previous study undertaken on a larger field (10 m × 15 m plot), in which we found that maize IE09S034 cultivation had no impact on the soil collembolan community^45^. Moreover, redundancy analysis also showed that the collembolan composition was not significantly correlated with the maize type. All these results indicated that Bt maize IE09S034 did not have adverse effects on soil Collembola. Although the results of previous studies have shown that the effect of Bt maize on soil Collembola varied with maize type and study sites, some studies have found that some species^46^ or groups^42^ were possibly influenced by Bt maize. However, most studies have reported that Bt maize had no adverse effects on soil Collembola in the field^45–48^. Studies undertaken in laboratories have reported that the consumption of Bt maize pollen^49^and leaf^50^ had no toxic effects on the representative Collembola *Folsomia candida* Willem, 1902. The results of previous studies have also indicated that Bt maize does not influence the diversity of other soil fauna^9,40,41,51^.

Interestingly, we found that the climate changes simulated by OTC had a significant effect on the soil collembolan communities, and the effects varied by the sampling stage. We showed that the soil humidity was significantly lower and the soil temperature was significantly higher in the OTC than outside at all sampling stages. Temperature and humidity are the two most important climate variables, and changes in these can affect the community structure and ecological function of soil fauna by affecting their growth, the environmental conditions of their habitat, and by regulating their “bottom-up” food sources^52,53^. The abundance and other diversity indices of Collembola were significantly higher in the OTC than outside at the seedling stage, but significantly lower in the OTC than outside at the heading and mature stages. This may be related to the physiological characteristics of Collembola, the seasonal changes in temperature and humidity in northeast China, and the root biomass of maize. The exoskeleton of soil Collembola is thin and poorly sclerotized^54^; thus, they are sensitive to soil temperature and humidity^28,29,55^. Our redundancy analysis also demonstrated that the collembolan composition was significantly correlated with the soil humidity and root biomass. In northeast China, the temperature was very low, usually < 0 °C before April. Under these conditions, the soil Collembola were basically dormant. Subsequently, as the temperature gradually rised, the soil Collembola began to revive and become active. Until the maize seedling stage (21th June, ~ 25 °C), the soil Collembola were in the recovery period. During this period, because the temperature inside the OTC was higher than that outside, soil Collembola recovered faster in the OTC than outside. Moreover, at this stage, the maize plant was still small, and the root biomass in the OTC was similar to that outside the OTC; therefore, the root exudate, which is the main food source of Collembola, was also not significantly different. Hence, the abundance and diversity of soil Collembola in OTC were higher than those outside OTC. Subsequently, from the seedling stage to the heading stage (4th August), the temperature continued to rise to ~ 35 °C, which is higher than the optimal living temperature for Collembola^56^, and the humidity in OTC (~ 25%) became significantly lower than the outside (~ 35%). This restricted the growth and development of Collembola^55,57^. Further, at this stage, the root biomass in the OTC was less than that outside. Therefore, the collembolan abundance and diversity in the OTC at the heading stage were significantly lower than those outside. At the mature stage (8th October), although the temperature had gradually decreased to the suitable temperature for Collembola (~ 18 °C), the humidity (~ 11%) and root biomass inside the OTC were still low. Consequently, along with the lower abundance and diversity of Collembola in the OTC at the heading stage, their reproduction was probably less than that outside; thus, the collembolan abundance and diversity in OTC were still significantly lower than those outside. Our results indicate that warming combined with drought influenced soil Collembola as follows: when the temperature is low, the soil collembolan abundance and diversity will be promoted by warming, and when the temperature is high and the humidity is low, soil collembolan abundance and diversity will be inhibited by warming combined with drought. Previous studies have also shown that the response of soil fauna to warming and drought depended on the ambient climate conditions^58^. For example, Harte et al.^59^ found that warming increased the micro arthropod biomass in a cold, wet summer but reduced the micro arthropod biomass in the following warm, dry summer. Climate change resulting in frequent summer droughts would probably decrease the abundance and diversity of forest soil fauna^60^, while extreme winter warming events have been shown to favor large-bodied, litter-dwelling Collembola^61^.

However, no effects of the interactions of the maize type with the OTC were detected on these parameters. Some previous studies have found that warming and drought can alter the ingredients and quantity of plants^16,24^, which may, in turn, affect the underground ecosystem by changing the organic matter input. Here, we found that the root biomass of maize was significantly altered when the ambient conditions changed to reflect those anticipated with climate change. However, this alteration had no relationship with the maize type, and no effects of the interactions of the maize type with the OTC or the maize type with the OTC and the sampling stage on Collembola were found. This result conflicts with our hypothesis that climate changes simulated by OTC would alter the effects of Bt maize on soil Collembola, indicating that warming combined with drought did not alter the effect of Bt maize on soil Collembola.

In summary, we demonstrated that the effects of climate change on soil Collembola depended on the ambient climatic conditions due to the thin and poorly sclerotized exoskeleton of soil Collembola: when the temperature is low, Collembola are promoted by warming, and when the temperature is high and the humidity is low, Collembola are inhibited by warming combined with drought. Bt maize had no significant effect on the soil Collembola, and the effect could not be altered by the climate change simulated by OTC.

## Methods

### Ethics statement

Field experiments were conducted in Jilin Province in 2017, which were approved by the Ministry of Agriculture and Rural Affairs of China. In this study, all methods were performed in accordance with the relevant guidelines, no vertebrates were included and none of the species are endangered or protected.

### Maize materials

Bt maize (transgenic *cry1Ie* maize hybrid IE09S034, which showed high toxicity to Asian corn borer) and its near-isogenic non-Bt maize Zong 31 (Z31), were provided by the Institute of Crop Sciences, CAAS, were used.

### Experimental design

The experiment lasted for 3 years, however, soil samples were only taken in the third year. It was carried out at the National Centre for Transgenic Plants Research and Commercialization/Jilin Academy of Agricultural Sciences in Gongzhuling (43°30′N, 124°49′E), Jilin Province, China. The mean annual temperature in this area is about 6.9 °C, and the mean annual precipitation is about 553 mm. The soil at the experimental field was the typical black soil of north-east China, with an alkali solution nitrogen content of 77.5 ± 0.1 mg/kg, organic matter content of 27.1 ± 0.1 g/kg, rapidly-available potassium level of 154.1 ± 0.8 mg/kg, soil available P level of 10.7 ± 0.1 mg/kg, and pH value of 5.4 ± 0.1. Sixteen experimental plots were arranged in a complete randomized block design with four replicated blocks. Four treatments, Bt maize in OTC (Bt-IN), non-Bt maize in OTC (Z31-IN), Bt maize outside OTC (Bt-OUT), and non-Bt maize outside OTC (Z31-OUT), were applied in each replicated block. Climate changes were simulated using OTC (4.2 m in diameter and 2.4 m in height) and plots without the framework of the OTC installed on the field were used as controls.

Maize seeds were manually sown on the 10th May in 2015 and 2016, and 16th May in 2017, withered maize stalks were manually removed in mid-April in 2016 and 2017. A distance of 20 cm between two adjacent maize plants in the same ridge, and a distance of 50 cm between two adjacent ridges were kept. Adjacent plots contained maize of different types. The same plot was used for planting the same maize type for the 3 years. No insecticides were applied during the study.

A humidity/temperature automatic recorder (Em50 Digital Data Logger, Decagon Devices Inc., Pullman, WA, USA) was placed in each plot to monitor the soil temperature and humidity of the 5 cm layer (recorded once per hour) in 2017. On each sampling day, at each sampling time (6 A.M., noon, and 6 P.M.), the soil temperature and humidity were measured using temperature and humidity recorders, and the mean of the three sampling times was used as the final data of the day.

### Sample collection

Samples were collected at three crop stages in 2017: seedling (21th June), heading (4th August), and mature stages (8th October). Four sampling points within each plot were randomly selected for each sampling time, from where ~ 200 mL of soil was collected in a plastic bag using a soil auger (15 cm in diameter, 10 cm in height). Simultaneously, the maize plants near the sampling points were dug out and the roots were cut off. Following this, the soil and the roots were transported to the laboratory.

The 200 mL soil sample was placed on a 20-mesh screen over a Macfadyen extractor funnel^62^, and the soil Collembola were extracted for 7 days at room temperature (20–25 °C). The soil Collembola moved down through the funnel and dropped into a collection bottle below containing 95% ethanol. The number and species of the Collembola were counted using a microscope (Olympus SZ51, Japan) at a magnification of about 40×. The Collembola were identified to species based on previously published literary sources^63–66^ using a microscope (Nikon 80i, Japan) at a magnification of about 200×. The roots were washed and dried in an oven at 55 °C for 48 h. The root biomass was then measured and the mean biomass of the four roots from one plot was used as the final data of the plot.

### Statistical analysis

Dominance (D) of soil Collembola is the proportion of individuals belonged to the *i*th species in the samples of the same treatment. Species with D > 10% were considered as numerically dominant^67,68^. The collembolan abundance and diversity parameters (species richness, Shannon–Wiener index, and Pielou’s evenness index) were calculated using Data Processing System software (version 2005, China). The Shannon–Wiener index (*H´*) and Pielou’s evenness index (*J*) were calculated as follows:

$$H^{\prime} = - \sum\limits_{i = 1}^{s} {P_{i} } \ln \left( {P_{i} \,} \right)$$

where *P*_*i*_ is the proportion of individuals belonged to the *i*th species in one sample.

$$J = {{H^{\prime}} \mathord{\left/ {\vphantom {{H^{\prime}} {\ln \;S}}} \right. \kern-\nulldelimiterspace} {\ln \;S}}$$

where *S* is species number of the collected Collembola in one sample.

Three-way ANOVA was performed using SPSS (version 23, IBM, USA) to evaluate the effects of the maize type, OTC treatment, and sampling stage on the soil collembolan abundance and diversity (species richness, Shannon–Wiener index, and Pielou’s evenness index). The collembolan abundance was log_10_ (N + 1) transformed before the analyses to ensure normality and equal variances and Levene's test of equality of error variances and asymmetry were used to analyze the homogeneity of variance. Tukey's HSD post hoc test (α = 0.05) was used when significant differences between treatment means occurred. Moreover, a one-way ANOVA was performed to evaluate the effects of OTC on the soil collembolan abundance and diversity, and certain important environmental factors (soil humidity, soil temperature, and maize root biomass) for the same maize type at each sampling stage using SPSS.

A type of redundancy analysis (RDA) was performed using CANOCO (Microcomputer Power, USA) to study the relationships between the soil collembolan community and study variables (including soil humidity, root biomass, sampling stage, OTC, soil temperature, and maize type). A Monte Carlo permutation test with 499 permutations was used to assess the significance of the canonical axes in the multivariate RDA analysis.
